# Adverse Effects of Cancer Treatment in Patients With Cervical Cancer

**DOI:** 10.7759/cureus.54106

**Published:** 2024-02-13

**Authors:** Mahesh Palagudi, Sneha Para, Nagasree Golla, Krishna Chaitanya Meduri, Sai Praneeth Duvvuri, Yethindra Vityala, Deepika Chowdary Sajja, Ujwala Damineni

**Affiliations:** 1 Department of General Medicine, P.E.S. Institute of Medical Sciences and Research, Kuppam, IND; 2 Department of General Medicine, Maheshwara Medical College and Hospital, Hyderabad, IND; 3 Department of General Medicine, I. K. Akhunbaev Kyrgyz State Medical Academy, Bishkek, KGZ; 4 Department of Research, AJ Research Centre, AJ Institute of Medical Sciences and Research Centre, Mangalore, IND; 5 Department of Pathology, International Higher School of Medicine, International University of Kyrgyzstan, Bishkek, KGZ; 6 Department of General Medicine, Santhiram Medical College, Tirupati, IND

**Keywords:** brachytherapy, chemotherapy, radiation therapy, adverse effects, cervical cancer

## Abstract

Background: In lower- to middle-income countries such as India, the literature on the adverse effects (AEs) of cancer treatment in patients with cervical cancer is very limited. This knowledge gap provides an opportunity to investigate and improve the quality of life for women with cervical cancer.

Objective: The purpose of this study was to assess the AEs of various cancer treatment combinations in patients with cervical cancer.

Methods: This observational, retrospective study analysed 1,030 women with cervical cancer, with a descriptive cross-sectional design, based on a review of medical records from patients who were followed up during the morbidity consultation conducted by a multidisciplinary team of doctors. The AEs of cancer treatment for women with cervical cancer were recorded in these medical records between October 14, 2019, and November 21, 2022, at 10 major public tertiary hospitals in India.

Results: This study analysed 1,030 women with cervical cancer aged between 21 and 80 years (mean age: 48.8 ± 13.9 years; p=0.30). Patients between the ages of 36-50 years reported the most AEs (30.2%; 95% confidence interval (CI): 29.1-32.8) among other age groups. Combined radiation therapy and chemotherapy (CT) was the type of cancer treatment in which there were more AEs, presenting in 56.0% (95% CI: 55-60.1) of patients. Adverse effects associated with the gastrointestinal system were observed in the majority (92.5%, 95% CI: 90.2-96.9) of cervical cancer patients.

Conclusion: Exposure to different cancer treatments, particularly combination therapy, induces AEs in patients during and after cervical cancer treatment.

## Introduction

Cervical cancer is the fourth most prevalent form of cancer among women globally [1], particularly among those between the ages of 30 and 60 [2]. Even though it is largely preventable, 604,127 women were diagnosed with cervical cancer in 2020 [3]. Cervical cancer claims a life every two minutes and is responsible for 341,831 deaths annually [3, 4]. Cervical cancer is the second most prevalent cancer among women in India, accounting for one-fifth of all cancer-related deaths worldwide. Compared to other Asian nations, the five-year relative survival rate is substantially lower, at approximately 46% (range: 34%-60%) [5].

Human papillomavirus (HPV) infection is a significant risk factor for cervical cancer, which predominantly spreads through direct skin-to-skin or skin-to-mucosal contact. Although sexual transmission is the most commonly reported mode of transmission, it is not the only means through which HPV can be transmitted. Other non-sexual methods of transmission include horizontal transmission of HPV between the fingers and mouth through skin contact [6]. Although patients are usually asymptomatic in the early stages, symptoms such as intermenstrual bleeding, postmenopausal bleeding, and foul-smelling vaginal discharge are not reported until the advanced stages of cervical cancer [7, 8].

The treatment for cervical cancer is similar to other types of neoplasms; however, patients are classified based on the 2018 International Federation of Gynaecology and Obstetrics (FIGO) uterine cervical cancer staging system [9]. While the use of cancer treatments may lead to adverse effects (AEs) when healthy tissues and organs are affected [10], these can differ for each person and vary according to the type of treatment.

The primary treatment for cervical cancer is radiotherapy (RT), which uses high-energy X-rays to target tumours and reduce cancer cell abundance [11, 12]. External beam RT, intensity-modulated RT, and brachytherapy (BT) are the three methods of radiation therapy currently used to treat cervical cancer [10]. Radiotherapy is a treatment procedure that causes short-term AEs such as fatigue, gastrointestinal changes (diarrhoea, nausea, and vomiting), dermatological changes, genitourinary changes (cystitis, and changes in the menstrual cycle), and haematological changes (anaemia, neutropenia, and thrombocytopenia). In the long term, genitourinary disorders such as vaginal stenosis, dyspareunia, rectal stenosis, chronic cystitis, haematuria, fistulas, and lymphedema have been observed with this treatment method [13]. Chemotherapy (CT) is another treatment option for cervical cancer that yields various AEs depending on the drug, dose, and duration of administration. The most frequent AEs include nausea, vomiting, hyporexia, hair loss, mucositis, and fatigue [14, 15].

Depending on the cancer stage, patients can undergo a combination of therapeutic procedures, which is defined as “combined therapy." However, combining CT and RT may alter the quality of life (QoL) of patients [16].

In lower- to middle-income countries such as India, the literature on the AEs of cancer treatment in patients with cervical cancer is very limited. This knowledge gap provides an opportunity to investigate and improve the QoL for women with cervical cancer. This study could help prevent and detect the occurrence of such AEs early by enhancing patient awareness of the need to continue the prescribed treatment, thereby reducing the frequency of discontinuation. The purpose of this study was to assess AEs associated with cancer treatment in patients with cervical cancer.

## Materials and methods

This observational, retrospective study analysed 1,030 women with cervical cancer with a descriptive cross-sectional design based on the review of medical records of patients who were followed up during the morbidity consultation conducted by a team of doctors. The AEs of cancer treatments for these women were recorded in their medical records between October 14, 2019, and November 21, 2022, at 10 major public tertiary hospitals in India.

The inclusion criteria were as follows: women pathologically diagnosed with squamous cell carcinoma, adenocarcinoma, or other or unspecified metastatic, persistent, or recurrent diseases, later receiving treatment, and presenting with AEs to non-surgical cancer therapies during the follow-up period. Women with cervical cancer who were referred by specialists for surgical treatment and those suffering from severe illnesses and mental disorders were excluded. Participants were selected through non-probabilistic sampling. Confidentiality was maintained regarding data collected from patients who provided their informed consent. This study was approved by the institutional ethics committee of Maheshwara Medical College and Hospital, Hyderabad (approval number: Maheshwara/Acd/IEC/Cert/009/2023), and was performed in accordance with the Declaration of Helsinki.

Information from the obtained data was consolidated in an anonymized database using licenced Microsoft Excel 2016 software (Microsoft Corp., Redmond, WA), and consecutive numbering was assigned to each patient. In this study, the following characteristics and variables were observed and recorded: sociodemographic metrics (age group, level of education, employment status, and monthly family income (USD)), clinical staging of cervical carcinoma (based on FIGO cancer staging system), cancer treatments, body mass index (BMI), histologic type, stage of cervical cancer, range per session in cancer treatment (therapeutic), gynecologic surgery history, adherence to treatment, and comorbidities. Organ systems were analysed according to the World Health Organization's adverse drug reaction monitoring register [17]. A reaction profile was created by calculating the number of records for each system or organ as a percentage of all reports.

All statistical analyses were performed using the Statistical Package for Social Sciences software version 15 for Windows (SPSS Inc., Chicago, IL). Data were presented as absolute frequencies (n) and percentages (%) with their respective 95% confidence intervals (CIs), and the mean age was presented as mean ± standard deviation. Pearson’s chi-square test was performed between the variables of cancer treatment and AEs grouped by organ system to determine statistically significant differences between the groups (p<0.05).

## Results

This study analysed 1,030 women with cervical cancer between the ages of 21-80 years (mean age: 48.8 ± 13.9 years; p=0.30). Participants in the age group of 36-50 years reported the maximum AEs (30.2%; 95% CI: 29.1-32.8) among age groups (Table 1).

**Table 1:** Sociodemographic characteristics of patients with cervical cancer showing adverse effects to cancer treatment N: number; ^a^mean ± standard deviation Statistical significance was defined as p<0.05.

Characteristics	N=1,030 (%)	95% CI	p-value
Age group			0.30
21–35 years	200 (19.5)	18.7–21.2	
36–50 years	310 (30.2)	29.1–32.8	
51–65 years	230 (22.4)	21.5–24.4	
66–80 years	286 (27.9)	26.8–30.3	
Age (years)^a^	48.8 ± 13.9 years		
Level of education			0.12
High school	772 (77.2)	72.5–81.9	
College	114 (11.1)	10.7–12	
Graduate	52 (5.0)	4.8–5.5.1	
Postgraduate	68 (6.7)	6.3–7.2	
Employment status			0.10
Full-time or part-time	221 (21.5)	20.7–26.2	
Not working	805 (78.5)	75.6–85.4	
Monthly family income (USD)			0.38
Very low (<100)	206 (20.1)	19.3–21.8	
Low (100–200)	363 (35.4)	33.7–41.5	
Average (200–500)	457 (44.5)	43.9–47.4	

In terms of the level of education, most of the patients studied up to high school (77.2%; 95% CI: 72.5-81.9). Regarding employment status, 78.5% (95% CI: 75.6-85.4) of the patients did not work, and 44.5% (95% CI: 43.9-47.4) of patients received monthly family income which was average (USD 200-500) (Table 1).

In terms of disease stage (according to FIGO uterine cervical cancer staging system), a total of 83.7% of the patients were classified as IB1, IIB, and IIIB. Patients with stage IIIB disease reported more AEs (35.7%; 95% CI: 34.5-39). The results of the analysis of the type of cancer therapy revealed that 56.0% (95% CI: 55-60.1) of the patients received combined RT and CT, and 15.8% (95% CI: 15.2-17.3) of the patients received combined RT, CT, and BT (Table 2).

**Table 2:** Clinical characteristics of patients with cervical cancer showing adverse effects to cancer treatments N: number; FIGO: The International Federation of Gynecology and Obstetrics Statistical significance was defined as p<0.05.

Characteristics	Level	N=1,030 (%)	95% CI	p-value
FIGO stages				0.17
	IB1	206 (20.0)	19.3–21.8	
	IB2	67 (6.5)	6.1–11.2	
	IIA	67 (6.5)	6.1–11.2	
	IIB	289 (28.0)	27.1–30.6	
	IIIA	39 (3.7)	3.6–4.3	
	IIIB	368 (35.7)	34.5–39	
	IVA	21 (2.1)	1.9–2.3	
Cancer treatments				0.04
	Radiotherapy	89 (8.6)	8.3–9.4	
	Chemotherapy	129 (12.5)	12.1–13.6	
	Radiotherapy and chemotherapy	576 (56.0)	55–60.1	
	Radiotherapy and brachytherapy	73 (7.1)	6.8–7.7	
	Radiotherapy, chemotherapy, and brachytherapy	163 (15.8)	15.2–17.3	
Body mass index				0.48
	Underweight	89 (8.6)	8.3–9.4	
	Normal	121 (11.8)	11.6–12.3	
	Overweight	357 (34.7)	33.1–37.8	
	Obesity	463 (44.9)	43.3–49.7	
Histologic type				0.03
	Squamous cell carcinoma	664 (64.4)	63.7–69.2	
	Adenocarcinoma	182 (17.7)	16.6–19.3	
	Other or unspecified	184 (17.9)	16.9–21	
Stage of cervical cancer				0.44
	Early stage	636 (61.7)	60.1–66.7	
	Advanced stage	394 (38.3)	36.7–49.2	
Range of sessions in cancer treatment				0.86
	1–9 sessions	69 (6.7)	64.7–73.2	
	10–19 sessions	128 (12.4)	12.1–13.8	
	20–29 sessions	321 (31.2)	30.5–32.4	
	30–39 sessions	512 (49.7)	48.7–54.2	
Gynaecologic surgery history				0.18
	Yes	692 (67.2)	64.2–73.8	
	No	338 (32.8)	31.735.2	
Adherence to medications				0.47
	Poor adherence	66 (6.4)	61.9-–70	
	Average adherence	210 (20.4)	19.7–22.8	
	High adherence	754 (73.2)	71.1–81.8	
Comorbidities				0.69
	Type II diabetes mellitus	206 (20.0)	19.1–23.8	
	Chronic kidney disease	21 (2.0)	1.9–2.8	
	Hypertension	147 (14.3)	13–15.5	
	Anemia	129 (12.5)	12.1–13.8	
	Coronary heart disease	63 (6.1)	5.9–6.8	
	Deep vein thrombosis	49 (4.8)	4.6–5.1	
	Rheumatoid arthritis	102 (9.9)	9.4–12.9	
	Other cancers	27 (2.6)	2.5–2.8	
	No	286 (27.8)	26.8–30.3	

Regarding BMI, 44.9% (95% CI: 43.3-49.7) of the patients were obese during the evaluation of AEs, and only 8.6% (95% CI: 8.3-9.4) of the patients were underweight.

Our results also showed that 49.7% (95% CI: 48.7-54.2) of the patients developed AEs received between 30 and 39 cancer treatment sessions, and 67.2% (95% CI: 64.2-73.8) of patients had at least one gynaecological surgical history (Table 2). Most of the patients (64.4%; 95% CI: 63.7-69.2) were diagnosed with squamous cell carcinoma, and 61.7% (95% CI: 60.1-66.7) of the patients exhibited early-stage cervical cancer.

Regarding treatment compliance, 73.2% (95% CI: 71.1-81.8) of the patients maintained good adherence, while 72.2% of patients had comorbidities, including type II diabetes mellitus (20.0%), hypertension (14.3%), anaemia (12.5%), rheumatoid arthritis (9.9%), and coronary heart disease (6.6%) (Table 2).

During the analysis of each AE in organ systems in patients with cervical cancer, AEs in the gastrointestinal system were observed in 92.5% (95% CI: 90.2-96.9) (Table 3), specifically, nausea in 19.0% (95% CI: 16.2-19.7), radiation enteritis in 17.1% (95% CI: 15.6-18.3), emesis in 16.4% (95% CI: 14.6-17.9), and diarrhoea in 14.7% (95% CI: 13.1-15.8) of the patients (Table 4).

**Table 3:** Characteristics of adverse effects from cancer treatments by organ systems in patients with cervical cancer N: number Statistical significance was defined as p<0.05.

Adverse effects on organ systems	Level	N=1,030 (%)	95% CI	p-value
Gastrointestinal				0.03
	Present	953 (92.5)	90.2–96.9	
	Absent	77 (7.5)	6.9–9.1	
Neurological				0.01
	Present	809 (78.5)	76.8–86.8	
	Absent	221 (21.5)	20.7–23.2	
Dermatological				0.04
	Present	756 (73.3)	71.9–81	
	Absent	274 (26.7)	25.4–28.5	
Genitourinary				0.01
	Present	732 (71.0)	69.6–75.3	
	Absent	298 (29.0)	26–31.1	
Other				0.09
	Present	199 (19.3)	16.8–21.1	
	Absent	831 (80.7)	78.8–83.6	

**Table 4:** Adverse effects of cancer treatment on the various organ systems of patients with cervical cancer N: number Statistical significance was defined as p<0.05.

Adverse effects on organ systems	Signs/symptoms	N=1,030 (%)	95% CI	p-value
Gastrointestinal		n=953		0.03
	Colitis	111 (11.6)	10.3–12.8	
	Diarrhea	140 (14.7)	13.1–15.8	
	Dyspepsia	88 (9.2)	8.4–9.5	
	Emesis	156 (16.4)	14.6–17.9	
	Radiation enteritis	163 (17.1)	15.6–18.3	
	Constipation	69 (7.2)	6.6–7.5	
	Epigastric pain	46 (4.8)	4.3–4.9	
	Nausea	180 (19.0)	16.2–19.7	
Neurological		n=809		0.01
	Adynamia	86 (10.6)	8.1–11.7	
	Asthenia	169 (20.9)	17.9–22.8	
	Headache	81 (10.0)	7.5–11.6	
	Pain	287 (35.5)	32.1–38.8	
	Insomnia	88 (10.9)	8.1–12.4	
	Paresthesia	50 (6.2)	4.5–7.4	
	Tinnitus	48 (5.9)	4.6–6.7	
Dermatological		n=756		0.04
	Dermatitis	180 (23.8)	18.7–24.2	
	Edema	80 (10.6)	7.2–11.7	
	Hyperpigmentation	236 (32.2)	27.7–34.2	
	Mycosis	32 (4.2)	2.7–4.8	
	Pruritus	138 (18.2)	17.7–19.2	
	Rash	90 (11.0)	8.5–12.4	
Genitourinary		n=732		0.01
	Actinic cystitis	36 (4.9)	3.3–5.7	
	Dysuria	88 (12.1)	8.6–14.3	
	Hematuria	69 (9.4)	6.4–10.7	
	Genital herpes	28 (3.8)	2.9–4.9	
	Urinary incontinence	29 (4.0)	2.9–3.1	
	Urinary tract infection	68 (9.3)	6.3–10.9	
	Leukorrhea	156 (21.3)	16.2–24.7	
	Polyuria	58 (7.9)	5.7–9.2	
	Vaginal ulcer	23 (3.1)	2.2–3.7	
	Vaginosis	177 (24.2)	18.7–25.6	

In this system, the type of cancer treatment for which there were more AEs was combined RT and CT, accounting for 58.3% of the patients (Table 5).

**Table 5:** Correlation between cancer treatments and adverse effects by organ systems N: total number of patients; n: number of patients who showed adverse effects by organ system *Pearson's chi-square test for categorical variables in cross-sectional studies (p<0.05)

Cancer treatments/adverse effects	Gastrointestinal		Neurological		Dermatological		Genitourinary		Other	
N=1,030	n=953	*p	n=809	*p	n=756	*p	n=732	*p	n=199	*p
Radiotherapy	75 (7.9)	0.03	59 (7.3)	0.01	46 (6.1)	0.05	41 (5.6)	0.04	12 (6.0)	0.03
Chemotherapy	116 (12.2)	0.04	97 (12.0)	0.08	69 (9.1)	0.04	43 (5.9)	0.03	19 (9.5)	0.02
Radiotherapy and chemotherapy	556 (58.3)	0.01	487 (60.2)	0.02	461 (61.0)	0.03	482 (65.8)	0.02	124 (62.3)	0.02
Radiotherapy and brachytherapy	59 (6.2)	0.05	52 (6.7)	0.04	52 (6.9)	0.04	48 (6.6)	0.05	21 (10.6)	0.04
Radiotherapy, chemotherapy, and brachytherapy	147 (15.4)	0.03	112 (13.8)	0.04	128 (16.9)	0.02	118 (16.1)	0.01	23 (11.6)	0.03

The second most common AEs were observed in the neurological system in 78.5% of the patients (95% CI: 76.8-86.8) (Table 3). The most common AEs observed in this system were pain in 35.5% (95% CI: 32.1-38.8), asthenia in 20.9% (95% CI: 17.9-22.8), and insomnia in 10.9% (95% CI: 8.1-12.4) of the patients after cancer treatment (Table 4). In this system, combined RT and CT was the type of cancer treatment with the most AEs in 60.2% of the patients (Table 5).

Similarly, dermatological AEs were observed in 73.3% (95% CI: 71.9-81) of the patients (Table 3). The most common AEs observed in this system were hyperpigmentation in 32.2% (95% CI: 27.7-34.2), dermatitis in 23.8% (95% CI: 18.7-24.2), and pruritus in 18.2% (95% CI: 17.7-19.2) of the patients (Table 4). In this system, combined RT and CT was the type of cancer treatment with maximum AEs in 61.0% of the patients (Table 5).

Finally, among the categorization of AEs by organ system, the genitourinary system had the highest prevalence at 71.0% (95% CI: 69.6-75.3) (Table 3). The most common AEs observed in this system were vaginosis in 24.2% (95% CI: 18.7-25.6), leucorrhoea in 21.3% (95% CI: 16.2-24.7), and dysuria in 12.1% (95% CI: 8.6-14.3) of the patients (Table 4). In this system, combined RT and CT was the type of cancer treatment for which there were more AEs in 65.8% of the patients (Table 5).

Adverse effects on the gastrointestinal, neurological, dermatological, and genitourinary organ systems, were significantly associated with the types of cancer treatments (p<0.05) (Table 5).

## Discussion

In this study, the average age of patients with cervical cancer who received treatment was 48 years, which corresponds to a Brazilian study showing that the average age of patients with cervical cancer was 45 years. In this study, 78.5% of women did not work. These data also correspond with those of a Brazilian study [18], in which more than 50% of the patients were unemployed. Similarly, in this study, most of them had incomes of up to one month’s minimum wage at 35.4% (95% CI: 33.7-41.5). These data show that cervical cancer is a disease that is prevalent in women who earn a low income, and the economic and social conditions decisively influence the timely diagnosis, access to treatment, and health conditions of patients [19].

Among women who received cancer treatment and developed at least one AE, 76% were classified as stage IIB and IIIB. This stage is characterised by tumour invasion of the pelvic wall and hydronephrosis [20]. In this study, the calculated BMI showed that 44.9% of the patients were obese and 34.7% were overweight. A recent meta-analysis showed that a higher BMI is clinically related to cervical cancer risk [21].

In this study, AEs associated with the gastrointestinal system were observed in the majority (92.5%) of patients, with the most common symptoms being nausea, diarrhoea, and emesis. These results coincide with a review of the efficacy of treatments for radiation-induced gastrointestinal toxicity, in which the QoL of half of the patients was affected by gastrointestinal symptoms (rectal bleeding or diarrhoea) [22]. Similarly, pelvic radiation disease, an outcome of disturbances in normal physiological functions, causes diarrhoea, tenesmus, incontinence, and rectal bleeding [23]. Diarrhoea is the most common toxicity experienced by patients during pelvic radiation [24]. However, this finding differs from that of a Swedish single-centre cohort study showing that intestinal toxicity is present in only 13.8% of patients [14].

Adverse effects associated with the neurological system were observed in 71% of patients, with pain being the most frequent. This result was similar to a previous prospective QoL analysis showing that several cancer treatment-related symptoms tend to develop soon after treatment and persist over time, including peripheral neuropathy, menopause, diarrhoea, and impaired sexual functioning [25]. Similarly, a systematic review reported pain and neuropathy as AEs in oncological therapies [26].

Adverse effects associated with dermatological systems were observed in 68% of the patients, and more than 50% of the patients reported hyperpigmentation and dermatitis. These AEs often lead to dose reduction and/or treatment delays, which can affect patient survival and QoL [27].

However, regarding the genitourinary effects, vaginosis and leucorrhoea prevailed in this study. An Indian randomised clinical trial compared treatment with CT-RT and RT and demonstrated its toxicity [28]. The most affected organs were the vagina (25%), bladder (15%), rectum (7.5%), and small intestine (3%). Other AEs that can occur with this system are associated with pelvic RT, which has a persistent negative impact on female sexual functioning [29]. The most frequent problems are vaginal dryness, dyspareunia, variable degrees of vaginal stenosis, loss of libido, and orgasmic disorder [29]. Comparatively, the most common AEs were hyporexia, heartburn, and arthralgia, where even a loss of appetite is an AE as an outcome of cancer treatments [30].

This study had a few limitations. The need for a larger sample size with five to 10 years of follow-up is recommended. Similarly, the limited literature on adequate differentiation of concepts must be considered, as some organ systems and AEs are usually grouped together.

## Conclusions

Exposure to different types of cancer treatments, particularly combination therapy, generates AEs in patients during and after cervical cancer treatment. During follow-up, patients reported AEs in the gastrointestinal system, such as nausea, emesis, actinic enteritis, and diarrhoea, whereas those in the neurological system reported pain, asthenia, and insomnia. On average, 70% of the women developed between two and four AEs derived from different cancer treatments. Therefore, it is essential to develop therapeutic strategies that allow for greater tumour control with a high QoL. Adverse effects in organ systems such as the gastrointestinal, neurological, dermatological, and genitourinary systems were significantly associated with the type of cancer treatment (p<0.05).

It is recommended that research be strengthened in relation to the AEs generated by cancer treatment. Hence, it is possible to adopt a pharmacodynamic and radiological model for this type of event in which an interdisciplinary team can support decision-making regarding dose individualization using predictive simulation methods. In addition, the inclusion of a survival model would allow for the determination of treatment efficacy.

## References

[1] Bray F, Jemal A, Grey N, Ferlay J, Forman D (2012). Global cancer transitions according to the Human Development Index (2008-2030): a population-based study. Lancet Oncol.

[2] Shrestha AD, Neupane D, Vedsted P, Kallestrup P (2018). Cervical cancer prevalence, incidence and mortality in low and middle income countries: a systematic review. Asian Pac J Cancer Prev.

[3] Ferlay J, Colombet M, Soerjomataram I, Parkin DM, Piñeros M, Znaor A, Bray F (2021). Cancer statistics for the year 2020: an overview. Int J Cancer.

[4] Bray F, Ferlay J, Soerjomataram I, Siegel RL, Torre LA, Jemal A (2018). Global cancer statistics 2018: GLOBOCAN estimates of incidence and mortality worldwide for 36 cancers in 185 countries. CA Cancer J Clin.

[5] International Agency for Research on Cancer (2011). Cancer survival in Africa, Asia, the Caribbean and Central America: database and attributes. Cancer Survival in Africa, Asia, the Caribbean and Central America, IARC Scientific Publication No. 162.

[6] Petca A, Borislavschi A, Zvanca ME, Petca RC, Sandru F, Dumitrascu MC (2020). Non-sexual HPV transmission and role of vaccination for a better future (review). Exp Ther Med.

[7] Palmer JE, Gillespie AM (2010). Diagnosis and management of primary cervical carcinoma. Trend Urol Mens Heal.

[8] Lea JS, Lin KY (2012). Cervical cancer. Obstet Gynecol Clin North Am.

[9] FIGO Committee on Gynecologic Oncology (2014). FIGO staging for carcinoma of the vulva, cervix, and corpus uteri. Int J Gynaecol Obstet.

[10] (2023). Side effects of cancer treatment. https://www.cancer.gov/about-cancer/treatment/side-effects.

[11] Cohen PA, Jhingran A, Oaknin A, Denny L (2019). Cervical cancer. Lancet.

[12] Johnson CA, James D, Marzan A, Armaos M (2019). Cervical cancer: an overview of pathophysiology and management. Semin Oncol Nurs.

[13] American Cancer Society (2020 (2023). Radiation therapy for cervical cancer. https://www.cancer.org/cancer/cervical-cancer/treating/radiation.html.

[14] Möller S, Mordhorst LB, Hermansson RS, Karlsson L, Granlund U, Riemarsma C, Sorbe B (2020). Combined external pelvic chemoradiotherapy and image-guided adaptive brachytherapy in treatment of advanced cervical carcinoma: experience from a single institution. J Contemp Brachytherapy.

[15] Bhatla N, Berek JS, Cuello Fredes M (2019). Revised FIGO staging for carcinoma of the cervix uteri. Int J Gynaecol Obstet.

[16] Shankar A, Prasad N, Roy Sh, Chakraborty A, Biswas ASh, Patil J, Rath GK (2017). Sexual dysfunction in females after cancer treatment: an unresolved issue. Asian Pac J Cancer Prev.

[17] (2023). International monitoring of adverse reactions to drugs adverse reaction terminology. https://iris.paho.org/handle/10665.2/45719?show=full.

[18] Correia RA, Bonfim CV, Ferreira DKS, Furtado BMASM, Costa HVV, Feitosa KMA, Santos SL (2018). Quality of life after treatment for cervical cancer. Esc Anna Nery.

[19] Ginsburg O, Bray F, Coleman MP (2017). The global burden of women's cancers: a grand challenge in global health. Lancet.

[20] Šarenac T, Mikov M (2019). Cervical cancer, different treatments and importance of bile acids as therapeutic agents in this disease. Front Pharmacol.

[21] Poorolajal J, Jenabi E (2016). The association between BMI and cervical cancer risk: a meta-analysis. Eur J Cancer Prev.

[22] Frazzoni L, La Marca M, Guido A, Morganti AG, Bazzoli F, Fuccio L (2015). Pelvic radiation disease: updates on treatment options. World J Clin Oncol.

[23] Morris KA, Haboubi NY (2015). Pelvic radiation therapy: between delight and disaster. World J Gastrointest Surg.

[24] Klopp AH, Yeung AR, Deshmukh S (2018). Patient-reported toxicity during pelvic intensity-modulated radiation therapy: NRG Oncology-RTOG 1203. J Clin Oncol.

[25] Kirchheiner K, Pötter R, Tanderup K (2016). Health-related quality of life in locally advanced cervical cancer patients after definitive chemoradiation therapy including image guided adaptive brachytherapy: an analysis from the EMBRACE study. Int J Radiat Oncol Biol Phys.

[26] Atkinson TM, Ryan SJ, Bennett AV (2016). The association between clinician-based common terminology criteria for adverse events (CTCAE) and patient-reported outcomes (PRO): a systematic review. Support Care Cancer.

[27] Sanmartín O, Beato C, Suh-Oh HJ (2019). Clinical management of cutaneous adverse events in patients on chemotherapy: a national consensus statement by the Spanish Academy of Dermatology and Venereology and the Spanish Society of Medical Oncology. Actas Dermosifiliogr (Engl Ed).

[28] Misra S, Lal P, Kumar Ep S (2018). Comparative assessment of late toxicity in patients of carcinoma cervix treated by radiotherapy versus chemo-radiotherapy - minimum 5 years follow up. Cancer Treat Res Commun.

[29] Jensen PT, Froeding LP (2015). Pelvic radiotherapy and sexual function in women. Transl Androl Urol.

[30] Speck RM, Lenderking WR, Shaw JW (2017). Integrating the patient voice with clinician reports to identify a hepatocellular carcinoma-specific subset of treatment-related symptomatic adverse events. J Patient Rep Outcomes.

